# Family centered care of hospitalized children: A hybrid concept analysis in Iran

**DOI:** 10.15171/hpp.2017.37

**Published:** 2017-09-26

**Authors:** Mahboobeh Khajeh, Nahid Dehghan Nayeri, Fatemeh Bahramnezhad, Akram Sadat Sadat Hoseini

**Affiliations:** ^1^Department of Pediatric Nursing, Tehran University of Medical Sciences, Tehran, Iran; ^2^Nursing and Midwifery Care Research Center, Tehran University of Medical Sciences, Tehran, Iran; ^3^Department of Critical Care Nursing, Tehran University of Medical Sciences, Tehran, Iran

**Keywords:** Concept formation, Family nursing, Pediatric nursing

## Abstract

**Background:** Family centered care of hospitalized children (FCCHC) is a multidimensional concept, which is directly associated with the context and healthcare system. This study was conducted to analyze the concept of FCCHC in Iran.

**Methods:** This concept analysis was conducted with the use of hybrid model in 3 phases: a literature review in the theoretical phase, semi-structured interviews and descriptive observations in the fieldwork phase, and combination of the results of 2 previous phases in the final analytical phase.

**Results:** The 4 main themes extracted in theoretical phase included "family and healthcare professional participation", "information sharing with families", "family and healthcare professional relationship based on dignity and respect" and "individualized care of family".Moreover, 4 themes were emerged in the fieldwork phase, including "family as a nonparticipant visitor", "one-way education", "non-supportive interactions" and "non-specific care of family". In third phase with combination of the results of 2 phases, the final definition of the concept was presented.

**Conclusion:** FCCHC is a comprehensive care that is affected by human and organizational factors and requires full participation of staff and family, effective interaction with family, education and information sharing with them, and individualized care of each family. By knowing the dimensions of the FCCHC, we will be able to run our activities to provide facilities and features for its optimal implementation in Iran.

## Introduction

Children illness and hospitalization with different reasons is an inevitable event that can disrupt the natural process of child’s life and even affect his/her development.^1,2^ In this situation, parents are faced with high level of stress and anxiety^3^; They may feel confused, angry and sinful; and even demonstrate their frustration with inappropriate behaviors such as; restlessness, irritability and isolation.^4^ Results of articles published from 1942 to 1956 presented scientific evidence about the adverse effects of separation of mother and child.^5^ Also, the unpleasant effects of hospitalization on children’s psychological health was the most important reason that led to introduction of family centered care (FCC) as an important approach in the care of hospitalized children.^6^

FCC is such an important phenomenon that has been examined with different approaches such as paradigm,^7^ philosophy,^1,8,9^ theory^10^ or concept.^6,11-13^ FCC is considered the standard for pediatric healthcare by many clinical professions, hospitals, and healthcare groups; but despite widespread endorsement, FCC continues to be insufficiently implemented into the clinical practice.^14^ Different literature expressed some characteristics for family centered care of hospitalized children (FCCHC). In addition, the literature has listed positive and rarely negative consequences of FCCHC.^6,15,16^

Among studies that have investigated FCC of children, there are some that have directly analyzed this concept.^6,12,13,17^ Nevertheless, most of these studies are not related to the care of children in the hospital^12,17^ or have been conducted in the neonatal intensive care unit.^13^ The aforementioned analysis are just limited to the previous studies and have been conducted theoretically without entering the field work. In addition, in majority of studies only the perceptions and experiences of nurses and the hospital staffs have been examined and families have been ignored.^8,18^ No FCCHC concept analysis research has been done in Iran and all of the mentioned researches have been conducted in the context of western society that their definition of family and the healthcare systems and their functions are very different than Iran.

Since a concept finds its true meaning in the social context, and this meaning usually differ from one culture to another; it is essential for a concept analysis to consider the social and native conditions in which the concept occurs.^19^ We selected the hybrid model as the most appropriate approach for analyzing the FCCHC concept in the context of Iran based on the experiences of whole people involved, especially families; because this model of concept analysis allows for the inclusion of actual participant experiences in defining the concept.^20^ On the other hand the hybrid model combines theoretical analysis with empirical observation which is helpful since no empirical definitions have been written about this concept in Iran. This allows for a focus on the essential aspects of the FCCHC definition. With detailed analysis of the concept and clarifying its dimensions, it can be operational. Then by implementing the concept in the healthcare system, we would take steps towards promoting the health of children and family at the community level.

In this regard, present study was conducted to analyze the FCCHC concept, based on the hybrid model.

## Materials and Methods

In order to analyze the concept of FCCHC, the researchers used the hybrid model that was introduced by Schwartz-Barcott and Kim. This model includes 3 stages of theoretical, field work and analytical phases.^20^ The method relies on concept development and is executed through qualitative explorations of a phenomenon in its place of occurrence. In this method, certain approach is adopted in which, the theoretical and experimental methods are combined together and the final development of the concept is attained through induction and comparison in such a way that, a type of reduction is formed at the end.^21^

### Theoretical phase

In the theoretical phase, a literature review was conducted by getting help from credible international and Iranian databases like “ProQuest Dissertations & Theses”, “PubMed (MEDLINE)”, “Elsevier, Ovid, Wiley”, “Google Scholar”, “SID”, “RICeST”, “IRANDOC”, “MEDLIB”, “IRANMEDEX” and “Magiran”.

The search’s keywords included “family centered care”, “family nursing”, “family centered nursing”, “patient-centered care”, “children” and “child/pediatric”. The search covered all the articles published from 1980 to 2016.

Some of the questions that had to be considered in literature review included “What is the nature of FCCHC? How has FCCHC been defined? How is FCCHC measured? What is the influencing factors on FCCHC?”

Considering the inclusion criteria (literature in Persian or English language with related keywords in the title, abstract, or list of keywords) and exclusion criteria (unrelated issues such as FCC in the community or/and home; FCC of dying children; care of family in neonatal intensive care; long-term FCC of children with mental retardation, cerebral palsy and similar diseases that are not related to the time of hospitalization), as well as assessing the quality of resource, in total, 60 resources (38 English articles, 17 Persian articles, 3 English theses and 2 English books) were used for theoretical phase of concept analysis.

At this stage, the extraction and analysis of data, was performed using the textual content analysis. In this study, the text of each literature was carefully studied and any sentences, phrases and words that implied the definition and dimensions of FCCHC were identified and categorized in different categories according to their repetition, differences and similarities.

### Field work phase

In field work phase, purposeful sampling approach was used for selecting the participants and an attempt was made to comply with principles of maximum variation sampling. The participants consist of a number of children admitted to pediatric units and their parents and siblings; nurses and physicians working in the Tehran children medical center as well as the pediatric units of the Imam Hussein and Bahar Hospitals in the city of Shahrood that had the ability to express their experiences in Persian language, willing to take part in the study and consented to be interviewed.

General information of the 27 participants is provided in Table 1.

In this phase, qualitative data were collected through face to face, semi-structured interviews with asking open-ended questions. The primary questions were the same as in the previous phase and further questions were derived from the participants’ responses. For instance, here are some questions that were asked of nurses: “How is the family involved in the daily care of the hospitalized children? In what circumstances, will the family become involved in the care of children? What factor/s prevents the family from participating in the daily care of children?”

Each interview lasted an average of 30 minutes to an hour and its place depended on the participants’ preference.

Since there are many problems for interviews, especially with children, and also, in order to enrich the data, the non-participant observation method was used in the data collection. Overall, 28 observational notes were prepared and recorded based on the time and place.

At this stage, we were seeking information directly from those involved in the care of hospitalized children in the context of Iran. At the same time, we did not want the results of previous related studies and available theoretical perspectives to affect our analysis. Therefore, we used the conventional qualitative content analysis approach to analyze the data, as described by Graneheim and Lundman.^22^ After transcribing the 27 interviews, 28 explanatory observations and field notes verbatim, they were coded. First, more than 1500 primary codes were extracted. Duplicated codes were eliminated and the remaining codes categorized in different categories according to their repetition, differences and similarities through reductive classification.

It should be noted that the Word software 2016 was used for data management.

*Scientific rigor of field work data*

To ensure the scientific accuracy and validity of the data collected at this stage, following steps were taken: using more than one method for data gathering (interview, observation and field note), maximum variation during sampling (selecting participant from all those involved in the care of hospitalized children with maximum diversity), prolonged engagement with the data gathering and analyzing (allocating a lot of time to review and modify the codes for several times), peer checking (rechecking the meanings and codes of the interview with research colleagues) and member checking (rechecking the meanings and codes of the interview with the participant).

### Final analytical phase

In the final analytical phase, the codes and categories obtained from the field work phase were compared with the data obtained from the literature review in the theoretical phase, and finally, the common features of FCCHC concept were identified and the definition of the concept was presented.

## Results

### Results of the theoretical phase

Based on the literature review, the primary foundations of FCC, was formed in the 1960s.^5,14^ Most of the papers found at the theoretical phase were in fact reviews of the definition and conceptualization of FCCHC^6,11,16,23^; some had dealt with these concepts through expressing its importance from the views of the people involved^8,24-27^; and some brought examples of FCC,^28-30^ or available challenges of its implementaion.^31^

Based on the literature, an operational definition of FCCHC is “a holistic approach to health care decision-making between the family and healthcare provider about all of the care processes such as policy and program development, implementation, evaluation, education, and delivery of care”.

According to the literature, 4 main themes of FCCHC include:

*Theme 1. Family and healthcare professional participation*

In most advanced and wealthy countries, families are considered as experts on the child’s abilities and needs and works with the healthcare team at all levels of care.

*Theme 2. Information sharing with families*

The literature showed that, one of the dimensions of FCCHC is education and information sharing with families. Healthcare personnel share the complete and unbiased information about the child and his/her treatment process with family in order to help them participate in the care and decision-making effectively. They also listen to family, their choices and perspectives. In the other words, information sharing is a 2-way and mutual process.

*Theme 3. Family and professional relationship based on dignity and respect*

According to literature regarding FCCHC, the relationship between family and healthcare professional forms based on respect; healthcare providers listen to family, their choices and perspectives patiently and they also provide unbiased and useful information for emotional support of the family without any expectations.

*Theme 4. Individualized care of family*

Literature mentioned that, in FCCHC family backgrounds such as family knowledge, values, beliefs, culture and strengths, individuality and the needs of all family members should be considered in planning and implementation of care. As well as personnel provides affordable support to meet the individual and special needs of the family.

### Results of the field work phase

After finishing the content analysis of field work data 4 themes were formed:

*Theme 1: Family as a non-participant visitor*

This theme indicated that, most of the participants considered family members just as a visitor, not as an active collaborator in child care. This issue is so manifest about fathers of hospitalized children. They allowed to spend just 1 hour a day with their children; so there is no chance for father participation in child care.

Here is an expression of a mother in this regard: *“Nurses called me to bring my child for urinal catheterization, but they did not allow me to be with my child in the treatment room or help them within the procedures…”*

Here is a nurse expression: “*Most parents do not do the tasks that have been assigned to do properly; so I would prefer to care for the child without the involvement of the parents. Almost all of the works in pediatric unit is professional; what can family do?! They can only waste our time! We do not have enough time to spend on family sabotages!”*

*Theme 2: One-way education*

In the field work phase, there was a considerable attention to documentation of the family education in the patient file than informing the family. Often these educations were about the rules in the pediatric unit, personal hygiene and caring of children at home; it rarely happened that healthcare providers informed the family about the child’s condition and type of treatment.

A mother expression in this regard:* “A nurse asked if my child already had blood transfusion. I was shocked, as I didn’t know why she asked this question! I said I don’t know; is there any problem with my kid? As she (nurse) wrote things, she said No! We want to inject him some blood. I asked blood? Why? She just said, ask the doctor... The doctor had given this order, without informing me or asking me any questions first…”*

*Theme 3: Non-supportive interactions *

An issue that was repeatedly mentioned by the participants or obtained from observational field notes was inappropriate interaction with families. This theme revealed that, although some healthcare providers have a good behavior with families; but often family members were not supported by the personnel, and did not get enough information, and also some of their questions remain unanswered. As a result, they felt confused and lonely.

A mother expression in this regard:* “My kid has diabetes, her doctor said there is a new treatment called Gene Therapy?! And you should go to Royan Institute. The doctor just said that, and didn’t answer my questions. He said he didn’t have any time and left the room. What should I do? I’m worried about my child…”*

*Theme 4: Nonspecific care of the family*

This theme indicated that, hospitalized children are considered only as patients; and the focus of the healthcare providers is on treatment than care of family. Indeed, they overlooked the individuality of each family and special needs.

A mother expressed:* “We live in this city alone…, I have a 3-month-old girl at home…, someone should be in hospital with my son, but (nurses) don’t let my husband stay here…”*

### Results of the final analytical phase

In third phase of the concept analysis with combination of theoretical phase results and insights achieved in field work phase, the final definition of the concept was presented:

“FCCHC is a multi-dimensional and comprehensive care of the family, which its proper implementation in Iran requires full participation of staff and family, effective interaction with family, education and information sharing with them and individualized care of each family”.

## Discussion

FCCHC is defined as a multi-dimensional and comprehensive approach of care which addresses the entire needs of the family in hospital. In contrast with the findings of the literature review phase, the main themes emerged out of our analysis following the field work phase revealed that, such care has not met its optimal level in our country and there are many shortcomings in this regard.

In Iran, due to undesirable environment of the most hospitals and insufficient personnel’s attention to family, the families lose their motivation to get involved in child’s care. Other context based obstacle is restriction on unlimited presence of the fathers and other family members, due to cultural and structural limitations in pediatric wards. Furthermore, sometimes family presence is limited by some personnel during the invasive and non-invasive procedures. Literature indicated that, family members’ presence is essential to support them and facilitate their participation in child care.^24^

The field work results revealed that FCC and FCCHC are unfamiliar concepts in the Iranian medical education and healthcare system. Most of our healthcare providers have no understanding of the correct meaning of the FCC and believe it is restricted to family education. Although the doctors and nurses often fail to provide necessary information about treatment process or listen to the family concerns, they have a good performance in family education and the documentation.

But an expert should be aware that, when a child is hospitalized, it is necessary to interact with family members patiently and try to identify and meet their specific needs. Moreover, in order to an individualized care of family, healthcare professionals should pay attention to family’s opinions, preferences, knowledge, beliefs, values and cultural context. Unfortunately, many needs of families are not considered in our hospitals and families may have many concerns, such as financial problems, being away from relatives, leaving the job, lack of suitable residence and place of rest around the hospital, nutrition and hygiene and etc, in addition to child illnesses.

It is worth noting FCC is influenced by the views, values, perceptions and personal understanding of the healthcare professionals.^32^ Having teamwork skills, commitment and knowledge^33^ and positive outlook on FCC and family role, willingness to take responsibility and responsiveness, lead to desirable FCC.^6^

Therefore, FCC programs should be established in university curriculum to promote the knowledge and correct the students and personnel’s perspective and attitude. Hospitals must also have a plan for training employees about FCC.^24,32^ Furthermore, as mentioned in various literatures, organizational policy and guidelines are necessary for better implementation of FCC in pediatric units.^32,33^ In this regard, one of the most important tasks of the organization is to provide resources. It should be noted that, in addition to the financial resources for developing and equipping the pediatric units and hospital setting, organizations should also provide human resources to compensate the shortage of nursing; because the large number of patients, excessive workload and shortage of personnel are factors that lead to the ignoring of FCC principles. Previous studies have highlighted this point repeatedly.^26,34^

### Limitations of the study

One limitation of this study was lack of access to the full-text copies of a few papers. Another limitation of this study was the language barrier, and the use of literature just with English and Persian languages. FCCHC in a context based concept that needs to be studied from the perspectives of diverse cultures and contexts in order to provide a comprehensive definition.

## Conclusion

By applying the hybrid model, we defined the concept of FCC of hospitalized children in Iran, as no studies on this topic had been conducted before. This study gives an insight into FCCHC in Iranian hospitals and factors that could be effective in its proper implementation. It seems, it can increase the personnel’s knowledge about such care, improve clinical performance and encourage service provider and healthcare team to implement FCCHC. In addition, providing a clear definition of the concept can make students to get better acquainted with the concept, and train healthcare practitioner according to its principles. Also, dimensions of the concept can be used to develop a tool for evaluating FCCHC in our context.

The literature review revealed, FCCHC is a set of principles that have been accepted as a care standard in developed countries; and today we are far from it, due to existing structural constraints in our country. We hope that, policymakers and those responsible for our health system, try to execute FCC in children hospitals and we can see its effects on health promotion of children and families.

However, we believe that, to clarifying more details of the FCCHC concept within the social and cultural context of Iranian hospitals, further studies are needed.

## Authors’ contributions

MK made substantial contributions to conception and design of the work; acquisition, analysis and interpretation of data; provided intellectual content and contributed to drafting and critical revision of manuscript; wrote the first draft, and made significant contributions to all subsequent versions of the manuscript. NDN made substantial contributions to conception and design of the work; analysis and interpretation of data; provided intellectual content and contributed to critical revision of manuscript. FB made substantial contributions to interpretation of data, provided important intellectual content, and was involved in drafting the manuscript. ASSH contributed to critical revisions and provided important intellectual content.

## Ethical approval

This research was approved by the Ethics Committee of Tehran University of Medical Sciences with number IR.TUMS.REC.1395.2503 dated April 30, 2016. All the participants were informed about the study objective and a written informed consent was obtained from each of them.

## Competing interests

There are no conflicts of interest.

## Acknowledgments

This paper is a report of the qualitative part of a mixed method study conducted in Tehran University of Medical Sciences, for nursing PhD degree. We thank the Vice-Chancellor for Research of Nursing and Midwifery Faculty of the University. We also thank all those who participated in this study. This research was supported by school of Nursing and Midwifery affiliated to Tehran University of Medical Sciences.

**Table 1:** Characteristics of the participants in field work phase

**Participant**	**Number and gender**	**Average age**	**Educational status**	**Average time**
Child	2 Females/2 males	9	4 Students	Child hospitalization (day): 3.87
Family	7 Mothers	30	1 Illiterate, 1 Primary, 1 Diploma, 4 BS	Child hospitalization (day): 3.87
	2 Fathers	40	1 Diploma, 1 BS	
	2 Siblings (female)	14	2 Students	
Nurse	8 Females	35	5 BS, 3 MS	Work in pediatric units (year): 7.69
Physician	3 Females/1 males	42	1 MD, 2 pediatricians, 1 professor	Work in pediatric units (year): 10.50
